# Artificial Liver and Renal Support System for Cynomolgus Monkeys with Surgery-Induced Acute Renal Failure: A Preclinical Study

**DOI:** 10.1155/2018/7456898

**Published:** 2018-05-24

**Authors:** Lei Feng, Guolin He, Lei Cai, Chaoyi Fu, Yang Li, Jun Weng, Xiaolin Huo, Qing Peng, Yi Gao

**Affiliations:** ^1^Department of Hepatobiliary Surgery II, Guangdong Provincial Research Center for Artificial Organ and Tissue Engineering, Guangzhou Clinical Research and Transformation Center for Artificial Liver, Institute of Regenerative Medicine, Zhujiang Hospital, Southern Medical University, Guangzhou 510280, China; ^2^Institute of Electrical Engineering, Chinese Academy of Science, Beijing 100190, China; ^3^State Key Laboratory of Organ Failure Research, Southern Medical University, Guangzhou 510282, China

## Abstract

Renal dysfunction is one of the most common complications of liver cirrhosis and is associated with increased morbidity and mortality. However, no available technology can simultaneously support liver and renal function in these patients. The aim of this study was to evaluate the safety and efficacy of an artificial liver and renal support system in cynomolgus monkeys with surgery-induced ARF. The ARF model was established by ligature of bilateral renal arteries in eight cynomolgus monkeys, which were randomly divided into a treatment group (*n* = 4) and control group (*n* = 4). Biochemical indexes were determined before and after surgery. Blood endotoxin levels, biochemical indexes, and bacterial cultures were assessed at 0, 3, and 6 h during treatment. System pressures and vital signs were recorded at 1 h intervals. Pathological examination was performed after death. ARF was successfully established, based on significant elevation of biochemical indexes and pathological examination. The treatment group had significantly reduced biochemical indexes relative to the control group. Measurement of blood endotoxins and aerobic and anaerobic bacteria cultures indicated no bacterial growth. The system pressures and vital signs were stable during treatment. The results indicate that our support system for the treatment of cynomolgus monkeys with surgery-induced acute renal failure is safe and effective.

## 1. Introduction

Renal dysfunction is one of the most common complications of liver cirrhosis and is associated with increased morbidity and mortality [1, 2]. The most common etiologies of renal dysfunction are pre-renal azotemia, acute tubular necrosis, and hepatorenal syndrome (HRS) [3]. Although the pathogenesis of renal dysfunction in HRS is incompletely understood, renal vasoconstriction is the most common cause [4].

At present, the preferred treatments for HRS are hemodialysis and other renal replacement therapies, such as continuous veno-venous hemofiltration [1]. Artificial liver and renal support systems (ALRSS) could help patients with HRS who do not respond to medical treatment [5]. However, no available technology can simultaneously support liver and renal function in patients with HRS. Thus, it is necessary to develop a new system that can support and improve liver and renal function in these patients.

Dialysis is the most commonly used method for treatment of acute renal failure (ARF) [6]. There are several modalities of dialysis, including continuous renal replacement therapy (CRRT) [7], intermittent renal replacement therapy (IRRT), and peritoneal dialysis. CRRT is the major method used for treatment of ARF, mainly because it is associated with better hemodynamic tolerance [8].

In this study, we focused on the safety and efficacy of a novel artificial liver and renal support system (ALRSS). Our previous research examined the efficacy of this system in treatment of an animal model of acute liver failure (ALF) and indicated that our ALRSS significantly improved liver function and survival time [9, 10]. The primary aim of the present study is to evaluate the safety and efficacy of the ALRSS by measuring changes of serum indexes in cynomolgus monkeys with surgery-induced ARF using CRRT mode. This article presents data on the safety and efficacy of our ALRSS in this animal model of ARF.

## 2. Materials and Methods

### 2.1. Animals

Eight male cynomolgus monkeys, 6 to 9 years old and weighing 9–12 kg, were provided by Guangdong Landao Biological Technology Co. Ltd. (33 Guanghua Road, Huangpu District, Guangzhou, Guangdong, China) (Certificate of Conformity SCXK [Guangdong] 2014-0010) (Table 1). The monkeys were cared for in strict accordance with the Guide for the Care and Use of Laboratory Animals. Each animal was kept individually in a special iron cage under standard conditions and fed three times a day with free access to water. The experimental protocol was reviewed and approved by the Institutional Review Board of the Second Affiliated Hospital of Southern Medical University, Guangzhou, China (No. ZJYY-2014-GDEK-003).

### 2.2. ALRSS

**Figure 1:**
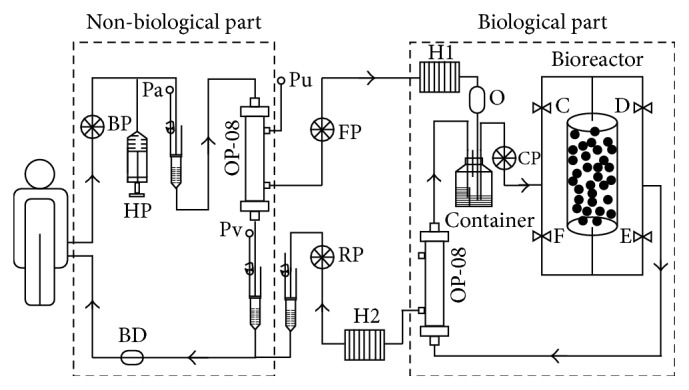
The schematic diagram of artificial liver and renal support system (ALRSS). BD, bubble detector; H1, H2, heater; C, D, E, F, pinch valve; BP, Blood pump; HP, heparin pump; FP, plasma pump; CP, circulating pump; RP, return pump; Pa, arterial pressure; Pv, venous pressure; Pu, ultrafiltration pressure; O, oxygenator; OP-08, membrane plasma separator.

The ALRSS includes a biological part and a non-biological part. The biological part is based on plasma perfusion of 4 trillion liver cells (such as C3A, CL-1 and primary porcine hepatocytes) which are seeded on microcarriers and placed in a bioreactor. They are separated from the patient blood by a non-biologic membrane (OP-08 membrane plasma separator) with a membrane pore diameter of 0.3 *μ*m. Non-biological part includes four pumps, heaters, heparin pump, pressure alarm, and bubble alarm, which can provide a variety of blood purification treatment modes (Figure 1).

### 2.3. Anesthesia and General Care

All monkeys were fasted for 12 h, but with free access to water, before surgery, and were placed under anesthesia to minimize suffering during the experiment. Anesthesia was induced by intramuscular injection of Zoletil 50 (Tiletamine 125 mg and Zolazepam 125 mg; Virbac, France) at a dose of 15 mg/kg body weight, followed by atropine (0.5 mg/kg). Then, the anesthetized animals were placed on an operating table that was equipped with warming equipment. After peroral endotracheal intubation, spontaneous breathing was maintained by continuous inhalation of isoflurane (1%-2%) and O_2_ (2 L/min), depending on the depth of anesthesia.

### 2.4. Establishment of the ARF Model

**Figure 2:**
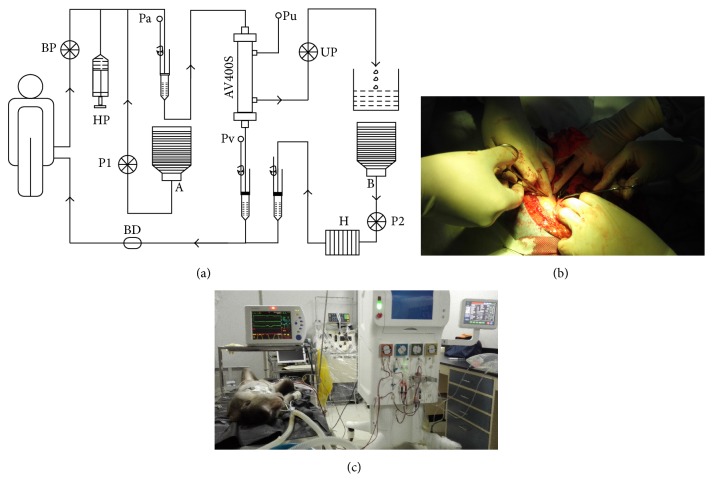
Continuous renal replacement therapy (CCRT) in the in artificial liver and renal support system (ALRSS) (a). Establishment of acute renal failure (ARF) in cynomolgus monkeys (b). ALRSS treatment (c). BP, Blood pump; HP, heparin pump; UP, ultrafiltration pump; BD, bubble detector; Pa, arterial pressure; Pv, venous pressure; Pu, ultrafiltration pressure; A and B, replacement fluids (see text); P1 and P2, replacement fluid pumps; H, heater.

After anesthesia, biochemical indexes were measured before surgery (baseline). A midline laparotomy was performed, and a 5 mm^3^ sample of renal tissue was removed for pathological examination (control tissue). First, the bladder was exposed, and an indwelling urinary catheter was inserted and connected to a closed collection bag to monitor urinary output. Then, the left and right renal arteries were dissected, separated, and ligatured using 2/0 silk thread (Figure 2(b)). After confirming that there was no obvious bleeding, the abdominal cavity was closed. To provide energy to the animals and protect against infection, 100 mL of glucose and sodium chloride with cefazolin sodium pentahydrate (60 mg/kg) was infused during surgery.

After surgery, each animal was placed in a special iron cage. Biochemical parameters were determined at 12 h and 24 h after surgery. The ARF model was considered to be successfully established when the serum creatinine (SCr) was more than 3 times the baseline level or above 354 *μ*mol/L, urine output was less than 0.3 mL/kg/h for 24 h or anuria was present for 12 h, or serum potassium was greater than 5.5 mmol/L [5, 11, 12].

### 2.5. Treatment Groups and Control Groups

The animals were randomly divided into two groups. Animals in the treatment group (*n* = 4) were anesthetized and treated by ALRSS for 6 h and those in the control group (*n* = 4) were anesthetized only for monitoring, with no treatment after the ARF model were successfully established.

### 2.6. Preparation of Replacement Fluids

The replacement fluid was formulated into two bags. Bag A contained saline (3000 mL), 10% KCl (24 mL), 25% MgSO_4_ (6 mL), 5% NaHCO_3_ (500 mL), and water for injection (750 mL). Bag B contained saline (3000 mL), 5% glucose solution (460 mL), 5% CaCl_2_ (50 mL), and water for injection (750 mL). The final molecular concentration of replacement fluid is glucose (13.8 mmol/L), Na^+^ (142.5 mmol/L), Ca^2+^ (2.38 mmol/L), Cl^−^ (112.5 mmol/L), HCO_3_^−^ (34.7 mmol/L), and SO_4_^2−^ (0.71 mmol/L). All solutions were formulated on a clean table under strict aseptic conditions. The replacement fluids were infused, half as pre-dilution (A) and half as post-dilution (B), through different pathways simultaneously during treatment (Figure 2(a)).

### 2.7. ALRSS Therapy

ALRSS therapy was given to the treatment group 24 h after establishment of ARF using CRRT mode, and the control group remained under anesthesia with no treatment. Animals in the treatment group were anesthetized again using the same method. To provide vascular access, a 7.5 Fr short-term dual lumen hemodialysis catheter (10 cm) was percutaneously placed in the right femoral vein, which was then separately connected to the arterial and venous ports of the ALRSS.

Before initiation of treatment, the pipeline and filter (AV400S UltraFlux filter-FMC) were washed with a solution of 0.9% saline and unfractionated heparin (5000 U/L). The extracorporeal volume accounted for approximately 30% of whole blood volume of each animal. To prevent complications from this large extracorporeal volume and to remove any circulating heparin not bound to the filter, additional priming was performed using a hydroxyethyl starch solution.

The blood flow rate was set at 32 mL/min and the ultrafiltration flow rate at 480 mL/h (48 mL/kg/h, 20%). Replacement fluids were infused, half as pre-dilution (A) and half as post-dilution (B). The flow rates of replacement fluid from pumps 1 and 2 were maintained at 4 mL/min (Figure 2(a)).

Anticoagulation was achieved by systemic administration of unfractionated heparin, and activated partial thromboplastin time (APTT) was monitored during treatment. An initial IV bolus of unfractionated heparin (180 IU/kg) was followed by continuous rate infusion beginning at 50 IU/kg/h and then titrated according to published guidelines to achieve a target APTT of 1.5- to 2.5-fold above baseline. At the end of the treatment, protamine sulfate was administered to prevent bleeding.

### 2.8. Vital Signs and Serum Biochemistry

Heart rate (HR), breathing rate (BR), oxygen saturation (SaO_2_), and mean arterial pressure (MAP) were recorded every 1 h during treatment. The presence of bleeding, high fever, and other serious adverse events were also recorded. Serum levels of creatinine (Cr), blood urea nitrogen (BUN), creatinine kinase (CK), lactate dehydrogenase (LDH), aspartate aminotransferase (AST), alanine aminotransferase (ALT), APTT, potassium (K), blood endotoxins, and bacterial cultures were determined at 0 h, 3 h, and 6 h during treatment. The system pressures were recorded every 1 h during treatment. Endotoxin levels were determined by Tachypleus Amebocyte Lysate (TAL) purchased from Sigma-Aldrich (Inc, USA).

### 2.9. Pathological Examination

All animals were sacrificed with a lethal intravenous injection of pentobarbital and KCl at the end of treatment (6 h from onset of CRRT/Sham). After death, a detailed autopsy was performed, and each animal's renal tissues were immediately fixed with formalin. Microscopy with hematoxylin and eosin (H&E) staining was used to assess renal necrosis and inflammatory cell infiltration. Microscopic changes of renal cells were also observed using electron microscopy (EM).

### 2.10. Statistical Analysis

The significance of differences between groups at different time points was determined using ANOVA, and differences between groups at the same time point were determined using student-*t* test. All data analyses were performed using SPSS version 21.0 statistical software (SPSS Inc, Chicago, IL, United States). A *p* value below 0.05 indicated significance.

## 3. Results

### 3.1. Establishment of the ARF Model

There were no significant changes in the vital signs of animals in either group before and after surgery. Twelve hours after surgery, there was evidence of oliguria (12 h urinary output less than 0.3 mL/kg/h). SCr increased as ARF progressed. Moreover, the serum levels of BUN, ALT, AST, CK, LDH, and potassium were significantly higher at 12 h than at baseline (*p* < 0.05 for all comparisons), indicating successful establishment of the ARF model.

### 3.2. General Conditions

The monkeys appeared to tolerate the treatment, and there was no evidence of neurological or respiratory complications. Moreover, there was no filter clogging, haemolysis, bleeding, or other events during treatment, although one monkey had errhysis from the incision.

### 3.3. Vital Signs

**Figure 3:**
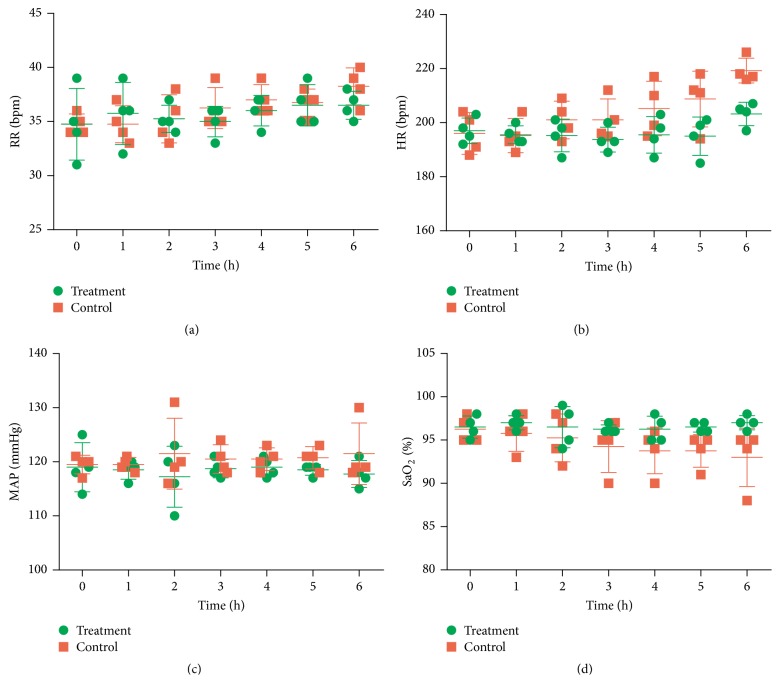
Changes in respiration rate (RR) (a), heart rate (HR) (b), mean arterial pressure (MAP) (c), and oxygen saturation (SaO2) (d) during the study period. Here and below in Figures 4 and 5, data are shown as means ± SDs (*n* = 4).

Figure 3 shows the changes in the respiratory rate (RR), heart rate (HR), mean arterial pressure (MAP), and oxygen saturation (SaO_2_) during treatment. The treatment group had slightly increased RR and HR, and slightly reduced MAP at the beginning of treatment compared with baseline, but these changes were not statistically significant. Except at 0 h and 1 h, the treatment group had significantly lower MAP than the control group. The SaO_2_ in treatment group was consistently above 95%, but the control group had SaO_2_ below 95% except at 0 h and 1 h.

### 3.4. Serum Biochemistry

**Figure 4:**
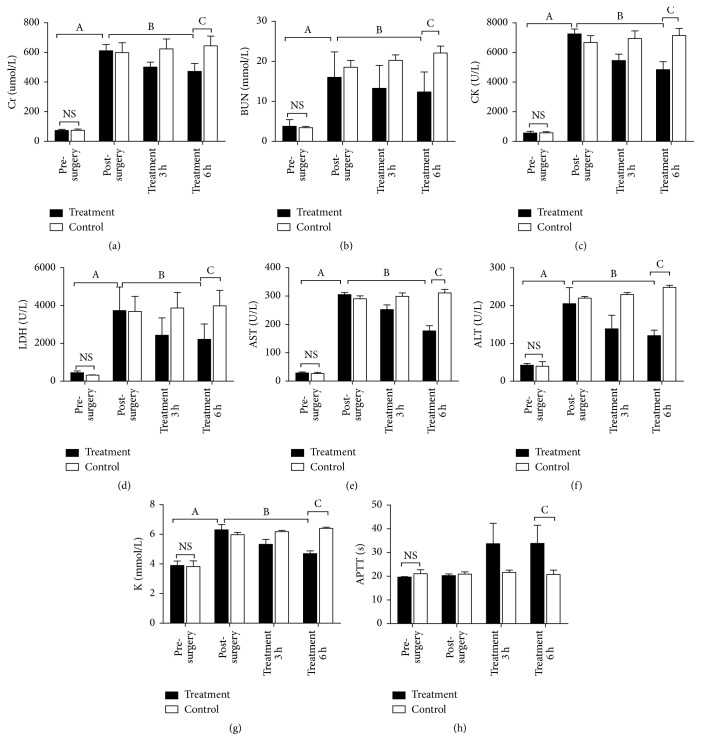
Levels of serum creatinine (SCr) (a), blood urea nitrogen (BUN) (b), creatinine kinase (CK) (c), lactate dehydrogenase (LDH) (d), aspartate aminotransferase (AST) (e), alanine amino transferase (ALT) (f), potassium (K) (g), and activated partial thromboplastin time (APTT) (h) in experimental animals before surgery, after surgery, after 3 h of treatment, and after 6 h of treatment. A *t*-test was used to determine the significance of differences. ^A^*p* < 0.01, before surgery versus after surgery in the treatment group; ^B^*p* < 0.05, after surgery versus 6 h of treatment in the treatment group; ^C^*p* < 0.01, treatment group versus control group after 6 h of treatment; NS, no significant difference.

Figure 4 shows the changes of serum biochemistry over time. These results show that the levels of SCr, BUN, CK, LDH, AST, ALT, and potassium were significantly higher after surgery. Moreover, the SCr, BUN, CK, LDH, AST, ALT, and potassium declined significantly after ALRSS in the treatment group, although the control group maintained elevated levels of these parameters. Injection of unfractionated heparin significantly increased the APTT in the treatment group.

### 3.5. System Pressure

**Figure 5:**
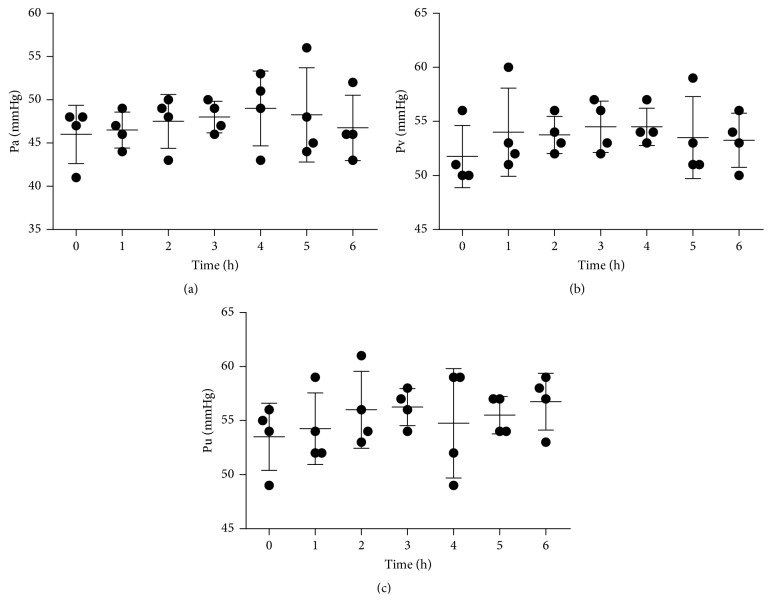
Changes of arterial pressure (Pa) (a), venous pressure (Pv) (b), and ultrafiltration pressure (Pu) (c) during the treatment period.

Figure 5 shows the pressure in different parts of the system. During treatment, the arterial pressure varied from 45 to 55 mmHg, the ultrafiltration pressure from 50 to 60 mmHg, and the venous pressure from 50 to 60 mmHg. The stability of these measurements and the lack of sudden changes indicated safe operation of the ALRSS.

### 3.6. Blood Endotoxins

Analysis of blood endotoxins showed the levels remained below 0.5 EU/mL. The aerobic and anaerobic cultures showed no bacterial growth after 7 days.

### 3.7. Animal Necropsy and Pathologic Changes

**Figure 6:**
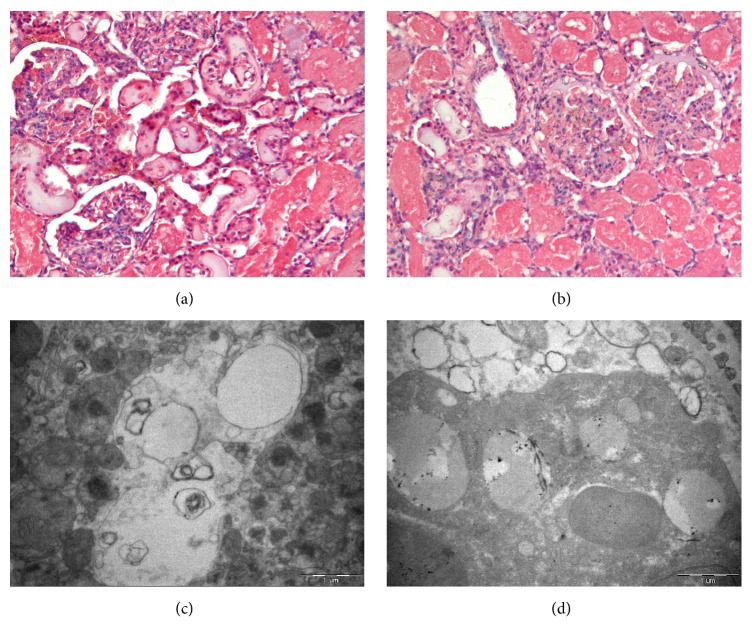
Light microscopy of representative renal necropsy specimens (H&E staining, ×100) in treatment group (a) and in control group (b); electron microscopy (×30,000) of a representative renal necropsy specimen in treatment group (c) and in control group (d).

Necropsy indicated no bloody ascites in any of the animals, but the kidneys were slightly narrow, hard, and blunt on the edges and were red or brown-gray in color. Light microscopy with H&E staining indicated tubular epithelial swelling and tubular necrosis. The proximal tubule epithelial cells were disintegrated, and there was evidence of cell necrosis, cell debris, and casts (Figure 6(a)). Electron microscopy indicated that although mitochondrial ultrastructure was unclear, there was significant swelling of the mitochondria and cell vacuolization (Figure 6(c)). The changes of renal HE and EM in control group were similar to treatment group (Figures 6(b) and 6(d)).

## 4. Discussion

Over the past several decades, a lot of artificial liver support systems have been developed to deal with ALF and ALF-related complications, such as HRS. The biological artificial liver support system included HepatAssist device [13], Extracorporeal liver assist device (ELAD) [14], Modular extracorporeal liver support (MELS) [15], Bio-artificial liver support system (BLSS) [16], and Amsterdam Medical Center-bioartifical liver (AMC-BAL) [17]. Although some of these devices were tested in clinical trials [13, 18], none has been approved by the FDA. The non-biological artificial liver support system has been widely used in intensive care units. However, several studies have shown that the non-biological system cannot significantly improve the survival time of ALF patients [19]. The only non-biological extracorporeal liver support system that showed an improvement in transplant-free survival time in patients with acute and acute-on chronic liver failure is plasmapheresis [20].

In this study, we tested an ALRSS in cynomolgus monkeys with surgically induced ARF. Our ultimate goal is to develop a system that could be used as bridge therapy for patients with ALF or ARF, while they are waiting for organ transplantation. The ALRSS is independently researched and developed by our group. Compared with other liver support system, our system can sequentially support liver and renal function in a single platform, and our system allows choice of a variety of blood purification treatment modes, such as hemodialysis (HD), hemofiltration (HF), plasma exchange (PE), continuous renal replacement therapy (CRRT), hemoperfusion (HP), high-volume plasmapheresis, and bioartificial liver support. Our previous research verified the efficacy of this system in treatment of ALF in an animal model [9, 10]. These previous results indicate that our ALRSS can significantly improve liver function and survival time.

Cynomolgus monkeys are increasingly used in biomedical research. We choose the cynomolgus monkey as a model animal because its structural, metabolic, biochemical, physiological, and immunological characteristics are similar to those of humans [21, 22]. These similarities make this species highly suitable for evaluation of our ALRSS in the treatment of ARF using CRRT mode. Pigs are commonly used animals for ALF test. However, the pig's physiological and biochemical characteristics are dissimilar from those of humans, and therefore the results from pigs are relatively poor for guiding clinical treatment.

Several methods can be used to establish an animal model of ARF, including the ischemia-reperfusion injury model, the renal artery ligation model, and the drug induction model [23–26]. Renal ischemia is a common cause of acute renal injury, so we used the surgical ligation of bilateral renal arteries to establish a cynomolgus monkey model of ARF. This method is rapid and has high reproducibility. The primary aim of the present study is to evaluate the safety and efficacy of the ALRSS by measuring changes of serum indexes in cynomolgus monkeys with surgery-induced ARF. So we think this model satisfied our primary aim.

To verify the safety of our ALRSS, we closely monitored the vital signs of cynomolgus monkeys and watched for adverse reactions during treatment. All monkeys tolerated the treatment well, and there were no serious complications, such as bleeding, clotting, allergic reactions, or high fever. We also tested for blood endotoxins and cultured blood samples. The blood endotoxin levels were below 0.5 EU/mL, indicating compliance with the standards for dialysate established by the Association for the Advancement of Medical Instrumentation (AAMI) [27]. In addition, the aerobic and anaerobic bacterial cultures showed no growth, indicating no evidence of blood infection. In addition, the whole pipeline and filter of the ALRSS were enclosed to ensure it remained uncontaminated.

System pressure is an important parameter of ALRSS because it can help identify emergency situations during treatment. Thus, if Pa increases significantly while Pv remains steady, it means the filter is clogged and needs to be changed. If the transmembrane pressure (TMP) increases beyond 600 mmHg, it could rupture, according to the specifications of the manufacturer. Thus, we monitored system pressure during the treatment. At all times, the system pressure remained stable, indicating that the system was operating safely.

The use of an anticoagulant is very important during extracorporeal blood circulation [28]. At present, the most commonly used anticoagulation methods are regional citrate anticoagulation [29] and systemic administration of unfractionated heparin [30]. We used unfractionated heparin for anticoagulation because it is inexpensive [31] and easy to use [32] and because protamine sulfate can be administered to protect from bleeding if there is an overload of unfractionated heparin, thereby improving safety. We also measured APTT throughout the treatment period, because this parameter can provide an indication of the need to adjust the titration of unfractionated heparin to achieve a target APTT of 1.5- to 2.5-fold above baseline [33].

We monitored the serum levels of Cr, BUN, CK, LDH, AST, ALT, and potassium throughout the treatment period to estimate the efficacy of the ALRSS. The treatment group had significantly reduced levels of these parameters after ALRSS. In contrast, the control group had slightly elevated levels of all these indexes. This indicates that our novel ALRSS was effective in the treatment of ARF in cynomolgus monkeys.

We also performed renal HE and EM after the animal died in the treatment and control groups. The result shows no significant difference between them, maybe because we used surgical ligation of bilateral renal arteries to establish the cynomolgus monkey model of ARF, and the injury was irreversible. Necropsy results showed no significant difference between two groups; the necropsy changes may be caused by surgical ligation of bilateral renal arteries.

This study is our first examination of the safety and efficacy of ALRSS in cynomolgus monkeys with surgery-induced ARF using CRRT mode. Thus, there were some limitations. First, we only assessed the efficacy of ALRSS by measuring changes of serum indexes. Second, we only compared the treatment group with an untreated control group, rather than with another treatment group. Third, the total time of treatment was only 6 h. In the future, we will further assess the safety and efficacy of our system by comparing it with other CRRT devices, by increasing the duration of hemodialysis (allowing prolonged monitoring of changes in serum indexes), and by analysis of survival time. In addition, the number of animals used was limited, and further studies with larger experimental groups are warranted to verify our results.

## 5. Conclusions

In conclusion, we successfully established an ARF model in cynomolgus monkeys and used an ALRSS that provided a safe and effective treatment. Further studies are needed to assess the role of the ALRSS in the treatment of ARF in this animal model and to develop guidelines for its clinical application.

## Figures and Tables

**Table 1:** Basic characteristics of the eight cynomolgus monkeys before surgery.

No.	Age (year)	Weight (kg)	Sex (F/M)	Groups (T/C)	BP (mmHg)	*T* (°C)	Cr (umol/L)	K (mmol/L)
1	6.5	9.8	M	T	116/68	37.2	78	3.7
2	8.0	10.1	M	C	111/76	37.1	69	4.1
3	8.5	9.7	M	T	108/79	36.7	70	3.6
4	7.5	9.4	M	C	107/58	36.8	78	3.9
5	6.0	9.5	M	C	122/64	36.9	85	3.7
6	7.5	11.3	M	T	124/71	37.5	80	4.1
7	8.5	10.7	M	C	112/74	37.8	65	3.4
8	7.5	10.6	M	T	123/60	36.5	63	4.2

F: female; M: male; T: treatment group; C: control group; BP: blood pressure; *T*: temperature; Cr: creatinine; K: Serum potassium.
